# GVI phospholipase A2 role in the stimulatory effect of sphingosine-1-phosphate on TRPC5 cationic channels

**DOI:** 10.1016/j.ceca.2011.06.003

**Published:** 2011-10

**Authors:** Eman AL-Shawaf, Sarka Tumova, Jacqueline Naylor, Yasser Majeed, Jing Li, David J. Beech

**Affiliations:** Multidisciplinary Cardiovascular Research Centre and the Institute of Membrane & Systems Biology, Faculty of Biological Sciences, University of Leeds, Leeds LS2 9JT, UK

**Keywords:** Cationic channel, Calcium-permeable channel, Transient receptor potential, Phospholipase, Sphingosine-1-phosphate, Lysophosphatidylcholine

## Abstract

The Transient Receptor Potential Canonical 5 (TRPC5) protein forms calcium-permeable cationic channels that are stimulated by G protein-coupled receptor agonists. The signaling pathways of such agonist effects are poorly understood. Here we investigated the potential for involvement of lysophosphatidylcholine (LPC) and arachidonic acid generated by group 6 (GVI) phospholipase A2 (PLA2) enzymes, focusing on stimulation of TRPC5 by sphingosine-1-phosphate (S1P) which acts via a pertussis toxin-sensitive (Gi/o protein) pathway without Ca^2+^-release. Experiments were on HEK 293 cells containing conditional expression of human TRPC5. Channel activity was recorded using an intracellular calcium indicator or whole-cell patch-clamp and PLA2 activity was detected using ^3^H-arachidonic acid. S1P stimulated PLA2 and TRPC5 activities. Both effects were suppressed by the GVI PLA2 inhibitor bromoenol lactone. Knock-down of GVI PLA2 by RNA interference suppressed channel activity evoked by S1P whereas activity evoked by the direct channel stimulator LPC was unaffected. Arachidonic acid did not stimulate the channels. Prior exposure of channels to LPC but not arachidonic acid suppressed channel activity evoked by S1P but not gadolinium, a putative direct stimulator of the channels. The data suggest roles of LPC and GVI PLA2 in S1P-evoked TRPC5 activity.

## 1 Introduction

Transient Receptor Potential Canonical 5 (TRPC5) is one of the seven mammalian TRPC proteins [1–4]. Like other TRPCs it is thought to form non-selective, Ca^2+^-permeable, cationic channels by organizing as a group of four proteins around a central ion pore in the plasma membrane. In growth cones TRPC5 occurs as a homomer [5] but it also exist as heteromers with other widely expressed TRPC proteins, including TRPC1 and its most closely related and functionally similar protein, TRPC4 [1,2]. Messenger RNA encoding TRPC5 has been most readily detected in the central nervous system but wider expression occurs and is relevant to a variety of organs including brain, heart and vasculature [2,6–8].

From the earliest studies it was recognized that TRPC5 and other TRPC channels are stimulated by G protein-coupled receptor agonists including ATP, acetylcholine, sphingosine-1-phosphate (S1P), glutamate and cholecystokinin [7–12]. In some cases the receptor has needed to be over-expressed with TRPC5 but all available data support the principle that many agonists acting through G protein coupled receptors enhance TRPC5 function. The effects are relevant to endogenous TRPC5-containing channels, for example in S1P modulation of vascular smooth muscle cell migration [8] and cholecystokinin modulation of fear responses in the amygdala [7]. Despite knowledge of the existence and importance of receptor stimulation of TRPC5, the signaling mechanisms mediating and regulating the process remain unclear. G protein activation is almost certainly a key step after receptor-activation, as supported by the observation that agonist effects are partially mimicked by intracellular GTP-γ-S (a stable analogue of guanosine triphosphate) and inhibited by GDP-β-S (a stable analogue of guanosine diphosphate) [8,10,13]. G proteins of the G_q/11_ type have been implicated [13,14] but the requirement is not absolute because activation by S1P or oxidized phospholipids is blocked by pertussis toxin, which inhibits G_i/o_ and not G_q/11_ [8,15]. In HEK 293 cells, although acting via different G proteins, endogenous muscarinic receptors and S1P receptors couple apparently similarly to TRPC5 [8]. S1P is an example of an agonist that activates TRPC5 and yet does not cause Ca^2+^-release in HEK 293 cells [8]. Similarly, oxidized phospholipids stimulate TRPC5 or TRPC1/5 channels without evoking Ca^2+^-release in HEK 293 cells or vascular smooth muscle cells [15].

It is also recognized that TRPC5 is a polymodal channel with a multiplicity of stimulators including extracellular lanthanides (e.g. gadolinium, Gd^3+^), extracellular or intracellular lysophosphatidylcholine (LPC), extracellular reduced thioredoxin and acidification [2,8,10,16–20]. Lanthanides appear to act directly via a glutamate residue near the ion pore, stabilizing the open state of the channels [16]. Therefore, stimulation of TRPC5 by lanthanides in the absence of other exogenous stimulators [12] may arise because openings of constitutively active TRPC5 channels are prolonged. LPC also appears to act relatively directly because it is effective in excised membrane patches and when G proteins are blocked [17].

Here we investigated if Group 6 (GVI) phospholipase A2 enzymes are relevant to TRPC5 function. Group 6 was selected because it shows particular tendency to generate LPC (and related lysophospholipids) and has been previously linked to agonist effects [21–25]. A commonly studied GVI PLA2 is GVIA (also called iPLA2β) but we were led also to investigate an additional member, GVIB (also called iPLA2γ). The study focused particularly on stimulation of TRPC5 by the receptor agonist S1P. For comparison we investigated effects of LPC and Gd^3+^.

## 2 Methods

### 2.1 Cell culture and TRPC expression

HEK-293 cells stably expressing tetracycline-regulated human TRPC5 have been described [12]. Cells were grown in DMEM-F12 medium containing 10% fetal calf serum, 100 U/ml penicillin and 100 μg/ml streptomycin. Cells were maintained at 37 °C in 95% air/5% CO_2_ and selected with 250 μg/ml zeocin and 10 μg/ml blasticidin. TRPC expression was induced by 1 μg/ml tetracycline (Tet+). Non-induced cells without addition of tetracycline were controls (Tet−). Cells were replated on poly-d-lysine-coated black 96-well plates (Corning or BD Biosciences) or 13 mm glass coverslips 24 h prior to experiments.

### 2.2 Ca^2+^ measurement

Induced (Tet+) and non-induced (Tet−) cells were plated at about 50% density in 96-well plates. Cells were incubated for 1 h in 2 μM fluo-4 AM in standard bath solution (SBS) at 37 °C. SBS contained (mM): 130 NaCl, 5 KCl, 8 d-glucose, 10 HEPES, 1.2 MgCl_2_ and 1.5 CaCl_2_; the pH was titrated to 7.4 with NaOH. Cells were washed three times with SBS at room temperature. The FlexStation II^384^ (Molecular Devices) was then used to measure [Ca^2+^]_i_ and changes in response to substance addition. SBS was the recording buffer and included a low concentration of gadolinium (Gd^3+^, 1 μM) to suppress background (non-TRPC5) channel activity. The FlexStation excited fluo-4 at 485 nm and collected emission at 525 nm. Readings were made every 10 s. Pre-treatments with 5 μM lysophosphatidylcholine, 25 μM bromoenol lactone or 10 μM arachidonic acid occurred for 30 min at room temperature prior to recordings. Control cells were incubated in the solvent (vehicle) only.

### 2.3 Whole cell voltage-clamp

Current recordings were made under voltage-clamp using the whole-cell configuration of the patch-clamp technique. Cells were investigated on coverslips at 20–30% density. The bath solution was SBS. The patch pipette solution contained (mM): 135 CsCl, 2 MgCl_2_, 1 EGTA, 10 HEPES, 5 Na_2_ATP and 0.1 Na_2_GTP; titrated to pH 7.2 with NaOH and filtered using a 0.2 μm filter (Sartorius, UK). For LPC experiments, LPC was added to the pipette solution at 20 μM or 0.1% methanol was the vehicle control; the two pipette solutions were compared alternately in paired experiments. The voltage paradigm was a 200 ms ramp protocol (−100 mV to +100 mV) applied every 10 s from a holding potential of 0 mV. Data were filtered at 2 kHz and digitally sampled at 4 kHz.

### 2.4 Short interfering (si) RNA

GVI PLA2 gene expression was down-regulated using a pre-designed siRNA sequence specific for GVIA PLA2 mRNA: 5′-GGAUCUCAUGCACAUCUCAtt-3′ (forward) and 5′-UGAGAUGUGCAUGAGAUCCtg-3′ (reverse); and for GVIB PLA2 mRNA: 5′-CAUAGUAAAUAGAGGGAUAtt-3′ (forward) and 5′-UAUCCCUCUAUUUACUAUGgt-3′ (reverse). HEK293-TRPC5 cells were allowed to reach ∼60% density on a 100 mm petri dish and then transfected with 1 μM GVIA PLA2 siRNA (Ambion, siRNA ID# 139141), GVIB PLA2 siRNA (Ambion, siRNA ID# s27074) or mock siRNA (Ambion, silencer^®^ negative control #1 siRNA). In brief, the transfection protocol was as follows: Cells were washed with 10 ml phosphate-buffered saline (PBS), harvested using trypsin and resuspended in DMEM-F12 medium. Cell suspension was transferred to a 15 ml tube and centrifuged at 200 × *g* for 10 min. Supernatant was decanted and the cell pellet resuspended in 0.2 ml transfection reagent (Amaxa Cell Line Nucleofector Kit V). To 0.1 ml, 100 μM solution of the appropriate siRNA was added (final concentration, 1 μM), mixed and transferred to a cuvette of a Nucleofector Kit V and submitted to nucleofection in an Amaxa device. Cells were then transferred to 6-well plates and incubated for 48 h. Cell selection antibiotics (blasticidin and zeocin) were excluded from culture medium at this stage. Transfection efficiency of 70–80% enabled functional assays using the FlexStation II^384^.

### 2.5 RT-PCR

Total RNA was isolated from HEK293-TRPC5 cells using a standard TriReagent protocol and treated with DNAse I (Ambion). 500 ng of this RNA was used for cDNA synthesis with oligo-dT primed AMV reverse transcriptase (+RT) and another aliquot was processed while omitting the reverse transcriptase (−RT). GVIA PLA2 PCR primer sequences were: forward 5′-TGCCATGACCGAGATCC-3′ and reverse 5′-CTCGCGGTGCTCATAGA-3′. GVIB PLA2 PCR primer sequences were: forward 5′-CGCAAGGGTGAGTATT-3′ and reverse 5′-ATATGGCACCTGTGCTTA-3′. Cyclophilin primers were: forward 5′-ACCCCACCGTGTTCTTCGAC-3′ and reverse 5′-TGGACTTGCCACCAGTGCCA-3′. TRPC5 primers were: forward 5′-GTCATCAAGCAAACGCT-3′ and reverse 5′-AGGCTAGAGGGCATTC-3′. Real time PCR reactions were performed with SYBR Green I on a LightCycler II (Roche, UK). Thermal cycling started with a 10 min hot start at 95 °C, then 40 cycles of: 95 °C (10 s); 55 °C (6 s); and 72 °C (16 s). At the end of PCR cycling, melting curve analysis was carried out by temperature ramping from 65 to 95 °C with a transition heating rate of 0.1 °C/s.

### 2.6 PLA2 activity assay

PLA2 in HEK293-TRPC5 cells was assayed by quantification of ^3^H-arachidonic acid released with or without stimulation [26,27]. Cells were seeded on poly-l-lysine coated 6-well plates and induced with tetracycline. On the next day, 0.5 μCi ^3^H-arachidonic acid (PerkinElmer) in 1 ml culturing medium was added to each well. After 24 h, cells were washed three times with PBS and kept for 30 min in the culture medium to recover. During this period, cells were preincubated with 25 μM BEL or the vehicle control (DMSO). After three washes with PBS, cells were stimulated for 20 min with 10 μM S1P or vehicle in HEPES-Tyrode's buffer (130 mM NaCl, 3 mM KCl, 0.8 mM KH_2_PO_4_, 0.8 mM MgCl_2_, 10 mM HEPES, 5.5 mM glucose, pH 7.4) containing 1.5 mM Ca^2+^. At the end of the incubation, fatty acid-free BSA (Sigma) was added to 0.3% final concentration to assist collection of released ^3^H-arachidonic acid. Liquid scinitaillation counting was used to determine the amount of released ^3^H-arachidonic acid in cleared medium and the total radioactivity from cell monolayers solubilised in 0.1% Triton X-100. For each sample the ^3^H-arachidonic acid release was quantified as the percentage of total radioactivity.

### 2.7 Chemicals and reagents

Dimethylsulphoxide (DMSO) was the solvent for 50 mM bromoenol lactone (BEL) and 15 mM (S)-BEL and (R)-BEL stock solutions. BEL compounds were from Cayman Chemical. Methanol was the solvent for 50 mM lysophosphatidylcholine (LPC) stock solution. Methanol was the solvent for 50 mM arachidonic acid (AA) and 10 mM sphingosine-1-phosphate (S1P) stock solutions. Water was the solvent for 100 mM gadolinium (Gd^3+^) stock solutions. Fluo-4 (2 μM) loading buffer included 8 μM pluronic acid (Invitrogen) and freshly prepared 2 mM probenicid. Stock solutions of 30% hydrogen peroxide (H_2_O_2_) and 200,000 U/ml catalase were prepared in SBS. For control experiments catalase was denatured by boiling it in solution for 10 min. Final concentrations of organic solvents were ≤0.06%, v/v. Unless specified, chemicals were from Sigma (UK).

### 2.8 Data analysis and presentation

Mean data are presented as mean ± standard error of the mean (SEM), where the *n* represents the number of independent experiments and the *N* represents the number of wells of a 96-well plate used in a single experiment. For patch-clamp experiments, *n* was the number of recordings from individual cells. ANOVA was used for statistical comparison between the population means and the unpaired Student *t*-test was used for comparisons between data sets. *P* values < 0.05 were taken as indicating significant difference. Data were analyzed and presented using Origin software (Microcal Inc., USA). Fluo-4 fluorescence (*F*) values are indicated as F* where * denotes that values were divided by 10^4^.

## 3 Results

### 3.1 Stimulation of PLA2 activity by S1P

**Fig. 1:**
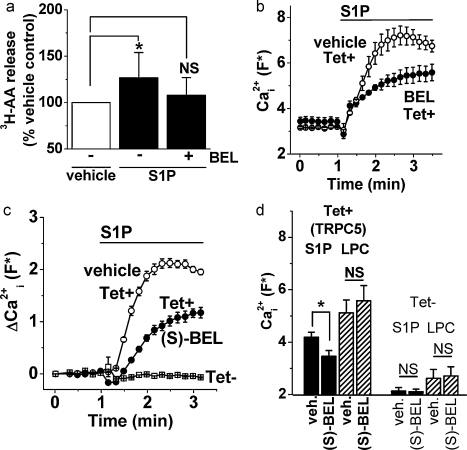
Inhibition of S1P-evoked PLA2 activity and TRPC5 Ca^2+^ entry by bromoenol lactone (BEL). (a) The iPLA2 activity was monitored as ^3^H-arachidonic acid (^3^H-AA) release in TRPC5 expressing HEK 293 cells treated with vehicle, 10 μM S1P or S1P after 25 μM BEL preincubation (*n*/*N* = 6/18). S1P significantly increased the AA release only in the absence of BEL. (b–d) Fluo-4 Ca^2+^ measurements from HEK 293 cells induced (Tet+) or not induced (Tet−) to express TRPC5. (b) Example effect of 25 μM BEL on the response to 10 μM S1P (*N* = 4). A small upward shift in the baseline is evident in the BEL group, which was statistically significant (*n*/*N* = 4/16). (c) Example effect of 10 μM (S)-BEL on the response to 10 μM S1P in Tet+ cells (*N* = 4). Shown for comparison is the lack of effect of S1P in Tet− cells (*N* = 4). (d) As for the example shown in (c) but the mean absolute Ca^2+^ measurement data for all experiments in the presence of 10 μM S1P or 5 μM LPC, without (veh., vehicle) or with (S)-BEL treatment, and with or without induction of TRPC5 expression (*n*/*N* = 4/16). (S)-BEL did not cause a significant shift in the baseline Ca^2+^ signal (*n*/*N* = 4/16).

There was no prior information on whether S1P stimulated PLA2 activity in HEK 293 cells and so the release of ^3^H-AA from metabolically pre-labeled cells was measured as an indicator of PLA2 activity. S1P caused a statistically significant increase in ^3^H-AA in HEK-TRPC5 cells, where as in the presence of the GVI PLA2 inhibitor BEL [28] there was no significant increase caused by S1P (Fig. 1a). The S1P data were further analyzed by normalizing the ^3^H-AA in the presence BEL to that in its absence (vehicle only); these two S1P groups were significantly different from each other (*P* = 0.031), confirming that BEL inhibited the S1P response. The data suggest that S1P stimulated GVI PLA2 in HEK 293 cells.

### 3.2 Inhibition of S1P but not LPC responses by bromoenol lactone (BEL)

TRPC5 activity was measured using an intracellular Ca^2+^ indicator (fluo-4) to detect Ca^2+^-influx in 96-well plate assays. Comparisons of different treatments were made within plates and, in each plate, cells induced (Tet+) and not induced (Tet−) to express TRPC5 were studied to determine the dependence on exogenous TRPC5 expression.

BEL in its racemic form inhibited TRPC5 activity that was stimulated by S1P (Fig. 1b). Separate experiments were performed with the (S) enantiomer of BEL which specifically inhibited the GVIA isoform of PLA2 at low micromolar concentrations [28]. (S)-BEL had a similar effect to that of racemic BEL, partially inhibiting TRPC5 stimulation by S1P (Fig. 1c and d). The effect of (S)-BEL was on the initial response to S1P over 2–4 min, conferring on the overall response a more sustained character (Supplementary Fig. I). There was no effect of S1P or (S)-BEL on Ca^2+^ in the Tet− (control) cells (Fig. 1c and d). The lack of effect of S1P in Tet− cells is consistent with prior work showing that S1P did not evoke Ca^2+^-release from intracellular stores in these cells [8]. LPC, a relatively direct stimulator of TRPC5 activity [17], evoked TRPC5-dependent Ca^2+^ entry that was resistant to (S)-BEL (Fig. 1d).

**Fig. 2:**
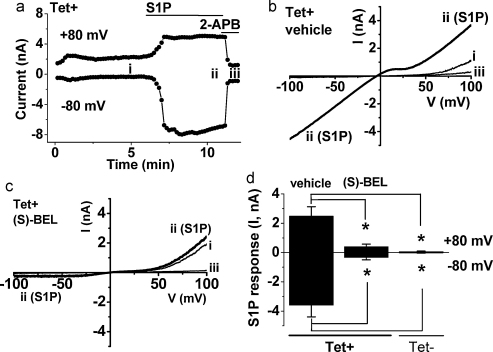
(S)-BEL sensitivity of S1P responses in whole-cell voltage-clamp recordings. All recordings were for HEK 293 cells induced to express TRPC5 (Tet+ cells) except for a subset of labeled data in (d). (a) An example time-series plot for a response to 3 μM S1P in the absence of (S)-BEL. Currents are shown for two points on the voltage ramp (−80 and +80 mV) and three time points are highlighted (i, ii, iii), which related to data in (b). 2-APB was applied at 75 μM. (b and c) Example current–voltage (*I*–*V*) relationships for three time points (see a): (i) before application of S1P; (ii) at the maximum response to S1P; and (iii) after inhibition by 2-APB. (b) Control cell pre-incubated in DMSO vehicle. (c) Test cell pre-incubated in 10 μM (S)-BEL. (d) Mean data for currents induced by 3 μM S1P at −80 or +80 mV in Tet+ (TRPC5) cells, recorded alternately after pre-incubation with DMSO vehicle (*n* = 6) or 10 μM (S)-BEL (*n* = 8). Shown also are data for the lack of response to S1P in Tet− cells that were not induced to express TRPC5 (*n* = 5).

The effect of (S)-BEL on S1P responses was also investigated under voltage-clamp in whole-cell patch-clamp recordings (Fig. 2). In vehicle control cells, S1P evoked large ionic currents that were abolished by the non-specific TRPC5 inhibitor 2-aminoethoxydiphenylborate (2-APB) (Fig. 2a) and showed the hooked shape in the current–voltage relationship (*I*–*V*) with a plateau between +5 and +25 mV, which is a characteristic of TRPC5 (Fig. 2b) [8,29]. The relatively linear *I*–*V* between −5 and −100 mV may be a consequence of activity-dependent gating [30,31] and is characteristic of large TRPC5-mediated currents evoked by S1P [8]. In the presence of (S)-BEL, there was basal TRPC5 activity but the activity evoked by S1P was small (Fig. 2c). Only recordings in which the characteristic TRPC5 *I*–*V* occurred were used for analysis. Non-induced (Tet−) cells showed no current in response to S1P (Fig. 2d) [8], confirming that currents were mediated by TRPC5. The mean data showed strong inhibition of S1P-evoked TRPC5-mediated current by (S)-BEL (Fig. 2d).

### 3.3 Inhibition of S1P but not LPC responses by GVI PLA2 knock-down

**Fig. 3:**
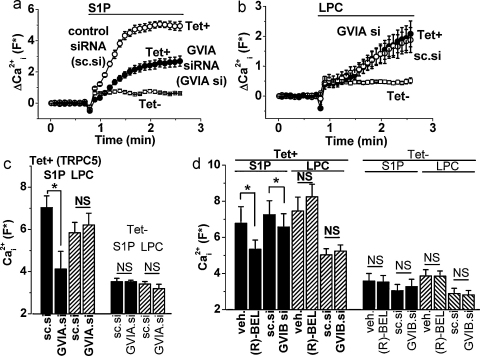
Effects of GVIA and GVIB PLA2 knock-down. Data were obtained by Ca^2+^ measurement after transfection of cells with short interfering (si) RNA (a–d) or (R)-BEL (d). (a and b) Example effects of GVIA PLA2 siRNA (GVIA si) on responses to 10 μM S1P (a) and 5 μM LPC (b) in TRPC5-expressing cells (Tet+), except where data without expression of TRPC5 (Tet−) are shown for comparison. Each data set is from *N* = 4. (c) Mean data for experiments of the type shown in (a and b) (*n*/*N* = 4/24 each). (d) As for (c) but for cells treated with 10 μM (R)-BEL in comparison to its vehicle (veh.) control (*n*/*N* = 4/16 each) or GVIB PLA2 siRNA (GVIB si) (*n*/*N* = 5/15 each). (c and d) LPC responses were measured 6 min after application of LPC.

To investigate the contribution of GVI PLA2 independently of BEL we used RNA interference to knock-down the expression of GVI PLA2 enzymes. Validation of the effectiveness and specificity of the knock-down is shown in Supplementary Fig. II. There was a lower density of cells after GVIA PLA2 knock-down (data not shown), consistent with the role of GVIA PLA2 in S-phase progression [32]. In an effort to compensate for this effect, cell number in test and control groups was matched prior to Ca^2+^-measurements. Similar to the effect of (S)-BEL, GVIA PLA2 siRNA partially inhibited TRPC5 activity evoked by S1P (Fig. 3a and c). In contrast, TRPC5 activity evoked by LPC was unaffected (Fig. 3b and c). There were no effects in Tet− (control) cells (Fig. 3c). These data support the hypothesis that GVIA PLA2 was involved in S1P-evoked TRPC5 activity.

As a further test of the specificity of (S)-BEL we investigated the other enantiomer (R)-BEL, which was described to inhibit the GVIB isoform but not GVIA PLA2 at low micromolar concentrations [28]. Perhaps surprisingly (R)-BEL inhibited S1P-evoked TRPC5 activity (Fig. 3d), suggesting contribution also of GVIB PLA2. To further investigate this suggestion we established specific knock-down of GVIB PLA2 by siRNA (Supplementary Fig. II). GVIB PLA2 siRNA caused modest but significant reduction in S1P-evoked TRPC5 activity without effect on stimulation by LPC (Fig. 3d). There were no effects of (R)-BEL or GVIB PLA2 siRNA on Ca^2+^ in Tet− (control) cells (Fig. 3d). The data suggest that GVIB PLA2 made a small contribution to S1P-evoked TRPC5 activity.

### 3.4 Inhibition of S1P but not Gd^3+^ responses by pretreatment with LPC

The initial products of GVI PLA2 are lysophospholipids and arachidonic acid. Therefore, GVI PLA2 may impact on TRPC5 by generating these lipid factors which then directly stimulate TRPC5. If this was the case, application of exogenous lipid might mimic and occlude S1P-evoked TRPC5 activity.

**Fig. 4:**
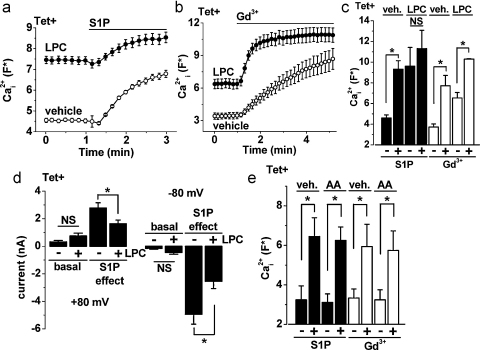
Effects of lysophosphatidylcholine (LPC) and arachidonic acid (AA) on S1P and Gd^3+^ responses. The panels show Ca^2+^ measurement (a–c and e) and whole-cell patch-clamp (d) data from HEK 293 cells induced to express TRPC5 (Tet+). (a and b) Example comparisons of 10 μM S1P (a) or 50 μM Gd^3+^ (b) effects in the presence of 5 μM LPC or vehicle control (a, *N* = 8 each; b, *N* = 6 each). (c) Mean data for the types of experiment shown in (a and b) (*n*/*N* = 3/15 and *n*/*N* = 5/20 for S1P and Gd^3+^ respectively). (d) Mean data for basal whole-cell currents sampled 10 min after break through to the whole cell (basal) and then evoked subsequently by 3 μM S1P (S1P effect). Current amplitudes were measured at −80 or +80 mV and recorded alternately with 20 μM LPC (+LPC, *n* = 12) or vehicle control (−LPC, *n* = 8) in the patch pipette solution. (e) Mean data as shown for (c) but where 10 μM arachidonic acid was used in place of LPC (*n*/*N* = 3/18 each).

LPC has non-specific detergent properties at high concentrations, preventing use of a concentration that generated maximum TRPC5 activity. Cells were, therefore, incubated with a concentration of LPC that stimulated TRPC5 but did not evoke non-specific signals in control (Tet−) cells. In Tet+ cells, LPC stimulated a rise in the intracellular Ca^2+^ concentration and suppressed the response to S1P (Fig. 4a and c). In order to determine if the LPC-evoked Ca^2+^ entry had simply saturated the Ca^2+^ indicator or maximized the total available TRPC5 activity we tested the effect of gadolinium (Gd^3+^), which is suggested to directly stimulate TRPC5 [16]. LPC did not suppress the Gd^3+^ response but increased its rate of onset (Fig. 4b and c).

To further investigate the effect of LPC pretreatment we used whole-cell patch-clamp recording, which enabled intracellular delivery of LPC. Recording with LPC in the patch pipette tended to yield spontaneous TRPC5 currents but the currents were not statistically larger than those in the control group (Fig. 4d). Responses to bath-applied S1P were, however, significantly reduced in cells recorded with LPC in the patch pipette (Fig. 4d).

It was previously reported that arachidonic acid had no effect on TRPC5 [17]. However, we re-investigated the effect of arachidonic acid in light of the new PLA2 data. Arachidonic acid failed to stimulate TRPC5 and had no effect of S1P- or Gd^3+^-evoked activity (Fig. 4e).

The data are consistent with lysophospholipids, exemplified by LPC, acting as second messengers coupling GVI PLA2 activity to TRPC5.

### 3.5 Enhancement of Gd^3+^ responses by GVI PLA2 inhibition

In contrast to S1P- and LPC-evoked TRPC5 activities, Gd^3+^-evoked TRPC5 activity was enhanced by GVIA PLA2 siRNA, GVIB PLA2 siRNA, BEL, (S)-BEL or (R)-BEL (Supplementary Fig. III), suggesting a fundamentally different property of this mode of TRPC5. We do not know the reason for the difference but we observed that catalase prevented the effect (Supplementary Fig. III), suggesting that endogenous H_2_O_2_ production was involved [29].

## 4 Discussion

The data of this study suggest that GVI PLA2 and LPC are components of a pathway that enables positive coupling between activated S1P receptors and TRPC5 channels. Both chemical blockade and knock-down experiments supported this conclusion. LPC, which was previously shown to be a relatively direct stimulator of the channels from the intracellular face of the membrane [17], could occlude at least part of the S1P response and it appeared to act downstream of GVI PLA2 because it stimulated the channels despite the inhibition of GVI PLA2. The data add to prior publications suggesting that Ca^2+^ channel regulation by PLA2s is important: TRPM8 channel function was linked to lysophospholipids and GVIA PLA2 because application of intracellular or extracellular lysophospholipid stimulated TRPM8 while stimulation of TRPM8 by store-depletion was suppressed by (S)-BEL [33,34]; and GVIA PLA2 was implicated in activation of endogenous non-selective cationic channels and voltage-gated Ca^2+^ channels [35,36].

There was quantitative discrepancy between the amplitude of the GVI PLA2-dependent S1P response in Ca^2+^ measurement and whole-cell patch-clamp experiments, with a stronger effect of PLA2 inhibition occurring in patch-clamp experiments (Fig. 2d cf. Fig. 1d). Patch-clamp is often considered to give a more direct measurement of channel activity, without membrane potential changes, and with less modulation of the net signal by other mechanisms such as Ca^2+^ uptake and extrusion. If this view is accepted, our data suggest that GVI PLA2 had a major role in coupling S1P receptors to TRPC5 channels. The Ca^2+^ measurement technique may have conferred amplification of small, residual, TRPC5 signals as Ca^2+^ accumulated in the cell and Ca^2+^ extrusion was attenuated as a consequence of the reduced driving force on Na^+^–Ca^2+^ exchange resulting from Na^+^ influx through the TRP channels [37]. Such effects may be pertinent to the observation that inhibition of GVI PLA2 changed the character of the S1P response in Ca^2+^ measurement experiments, giving the appearance of a reduced early but not late phase of the response (Supplementary Fig. I); conceivably the S1P-evoked activation of TRPC5 was strongly suppressed but prolonged activation of residual signal led to gradual accumulation of intracellular Ca^2+^.

Although our data support a role for GVI PLA2 and LPC in a pathway linking S1P receptors to TRPC5 channels it is premature to exclude a cooperative or permissive role of this system with other pathways. Our data do not exclude such additional pathways in the S1P response or responses occurring via other receptors. Direct G protein effects on the channels, for example, are not excluded. Also, in view of studies of other receptor-channel coupling mechanisms and widespread detection of channel sensitivity to PIP_2_ [38], a coupling mechanism that deserves consideration is PIP_2_ depletion as a consequence of receptor-activation of phospholipase C (PLC). A recent study identified SESTD1 protein as a partner of TRPC5 and showed that it bound PIP_2_ [39]. Such a mechanism would operate effectively for TRPC5 if: (i) the basal PIP_2_ concentration in unstimulated cells inhibited TRPC5; (ii) agonist-evoked PIP_2_ depletion was sufficient to cause de-inhibition of TRPC5; and (iii) TRPC5 had sufficient constitutive activity or there was concurrent presence of a TRPC5 activator that enabled de-inhibition to release TRPC5 channel activity. Unfortunately, a PIP_2_-dependent mechanism sits awkwardly with the available data which suggest that TRPC5 is stimulated, inhibited or unaffected by PIP_2_ [40,41] and the finding that a related channel (TRPC4β) is receptor-stimulated yet not PIP_2_ sensitive [42]. Intriguingly, although S1P did not evoke Ca^2+^-release, suggesting that it did not evoke inositol 1,4,5-triphosphate (IP_3_) production or PLC activity, the PLC inhibitor U73122 inhibited S1P-evoked TRPC5 activity [8]. The specificity of U73122 for PLC is, however, not certain. Even if the effect of U73122 was due to PLC inhibition, PLC may be a co-factor for TRPC5 function or be required for surface expression because we have found that U73122 also inhibited Gd^3+^-evoked TRPC5 activity.

Other signals associated with PLC have been investigated as potential intermediates in receptor-coupling to TRPC5 and these may be particularly relevant to receptor agonists that cause Ca^2+^-release, such as acetylcholine and histamine. These other PLC-related signals include increased concentrations of IP_3_, diacylglycerol (DAG) and Ca^2+^ (resulting from IP_3_-induced Ca^2+^-release). While one study suggested a role of IP_3_ in TRPC5 stimulation [13], others found no effect of IP_3_ or IP_3_ receptor blockade [10,11,43,44]. There is agreement that DAG does not stimulate homomeric TRPC5 [10,13,20,45]. Neither S1P nor LPC evoked Ca^2+^-release or other Ca^2+^ signals in HEK 293 cells in the absence of exogenous TRP channel expression [8,17], which not only suggested that PLC was not activated but that elevated Ca^2+^ was not the down-stream messenger coupling receptors to TRPC5. Ca^2+^-release events below our detection threshold cannot be excluded but it is notable that prior depletion of intracellular Ca^2+^ stores did not prevent S1P-evoked TRPC5 activity [8]. The data suggest that Ca^2+^-release is not critical in receptor-activation of TRPC5, but it could have importance, especially if the receptor agonist evokes Ca^2+^-release, repetitive Ca^2+^ entry, or inhibition of Ca^2+^ extrusion. Ca^2+^ has complex effects on TRPC5. We know it impacts on TRPC5 activity because buffering of intracellular Ca^2+^ below the basal physiological concentration depresses channel activity and elevated Ca^2+^ above the basal concentration is a potentiating co-factor, most notably with receptor agonists [46–48]. Therefore, intracellular Ca^2+^ almost certainly has a positive modulator role in physiological effects on TRPC5. In summary, DAG is excluded as a stimulatory signal coupling any receptor to TRPC5, and direct involvement of IP_3_ is unlikely. Roles of Ca^2+^ elevation and PIP_2_ depletion are likely in some contexts but neither seems to be a sufficient or singular event linking receptor to channel. There is no compelling evidence that S1P stimulates PLC in HEK 293 cells, despite it being a very effective stimulator of TRPC5. We conclude that PLC activation, IP_3_ and Ca^2+^-release are unlikely to be obligatory for receptor-activation of TRPC5.

Mechanistically it is unclear how catalase prevented the effect of GVI PLA2 inhibition on Gd^3+^ responses. We speculate that constitutive generation of H_2_O_2_ by mitochondria led to membrane lipid peroxidation, priming TRPC5 channels for low-level activity. Basal activities of GVI PLA2s may have then suppressed the process by removing peroxidized phospholipids [25,49]. The effect of the H_2_O_2_ on TRPC5 would have become apparent in Gd^3+^ responses because Gd^3+^ stabilized the open state of basally active channels. Other stimulators would not have been affected because they did not act by stabilizing the channel open state; that is, Gd^3+^ responses were proportional to constitutive (basal) activity of TRPC5, where as other responses were not. Alternative hypotheses can be generated because there are complex house-keeping roles of GVI PLA2s in maintaining membrane lipid integrity and TRPC5 is sensitive to a range of different lipid factors [20,50,51]. All of these suggestions are tentative and would required further investigation.

In summary, the study suggests that GVI PLA2 and LPC are components in the coupling mechanism between S1P and TRPC5 channels. Future studies will be required to determine the relevance of these findings to endogenous TRPC5-containing and related channels of the cardiovascular and nervous systems where lysophospholipids and PLA2 enzymes have important roles [52–56].

## Conflict of interest

None.
