# Supplementary material for: GVI phospholipase A2 role in the stimulatory effect of sphingosine-1-phosphate on TRPC5 cationic channels
Source: Cell Calcium. 2011 Oct;50(4):343–50. doi: 10.1016/j.ceca.2011.06.003 (PMC3195672; doi:10.1016/j.ceca.2011.06.003)
Supplement: Supplementary file 1 [file mmc1.pdf]

SUPPLEMENTARY INFORMATION

GVI phospholipase A2 role in the stimulatory effect of sphingosine-1-phosphate on TRPC5 cationic channels

Eman AL-Shawaf, Sarka Tumova, Jacqueline Naylor, Yasser Majeed, Jing Li & David J Beech

Multidisciplinary Cardiovascular Research Centre and the Institute of Membrane & Systems Biology, Faculty of Biological Sciences, University of Leeds, Leeds, LS2 9JT, UK.

Author for correspondence: Professor David J Beech, Institute of Membrane & Systems Biology, Garstang Building, Mount Preston Street, Faculty of Biological Sciences, University of Leeds, Leeds, LS2 9JT, England (UK). Telephone +44-(0)-113-343-4323; Fax +44-(0)-113-343-4228; Email d.j.beech@leeds.ac.uk

Supplementary Figure I. *Effect of (S)-BEL on long-duration S1P responses.* The figure shows an example effect of 10 µM (S)-BEL on the response to 10 µM S1P in Tet+ cells (N=4) during a response of 9 min duration.

Supplementary Figure II. *Validation and specificity of siRNA knock-down.* Mean data for quantitative RT-PCR analysis of cyclophilin (cyclo.), TRPC5, GVIB PLA2 and GVIA PLA2 mRNA species. For each species there was comparison of control and test (GVIA) siRNA treated groups (N=3 for each).

Supplementary Figure III. *Potentiation of Gd^{3+} responses by GVI PLA2 inhibition.* (**a**) Mean data for Ca^{2+} measurements in TRPC5-expressing (Tet+) or non-expressing (Tet-) cells showing effects of GVIA PLA2 siRNA (n/N=4/24), 10 µM (S)-BEL (n/N=4/16), GVIB PLA2 siRNA (n/N=5/15) and 10 µM (R)-BEL (n/N=4/16) on stimulation by 50 µM Gd^{3+}. The control siRNA is indicated as sc.si; veh. indicates the vehicle (solvent) control for the BEL experiments. (**b**) Ca^{2+} measurement data for TRPC5-expressing cells investigating effects 50 µM Gd^{3+} in the continuous presence of 500 U/ml catalase and after pretreatment with 10 µM (S)-BEL or vehicle control. The data are mean values for n/N=3/18 each.

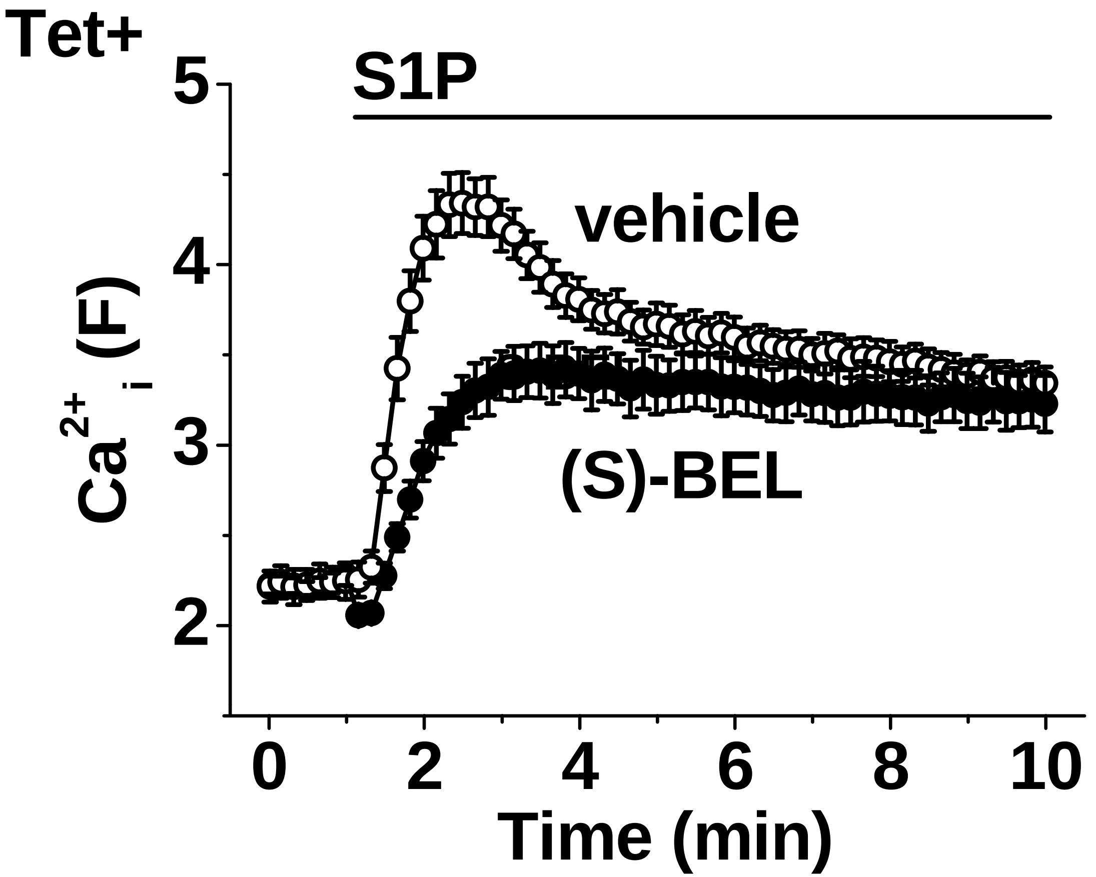

Supplementary Figure I

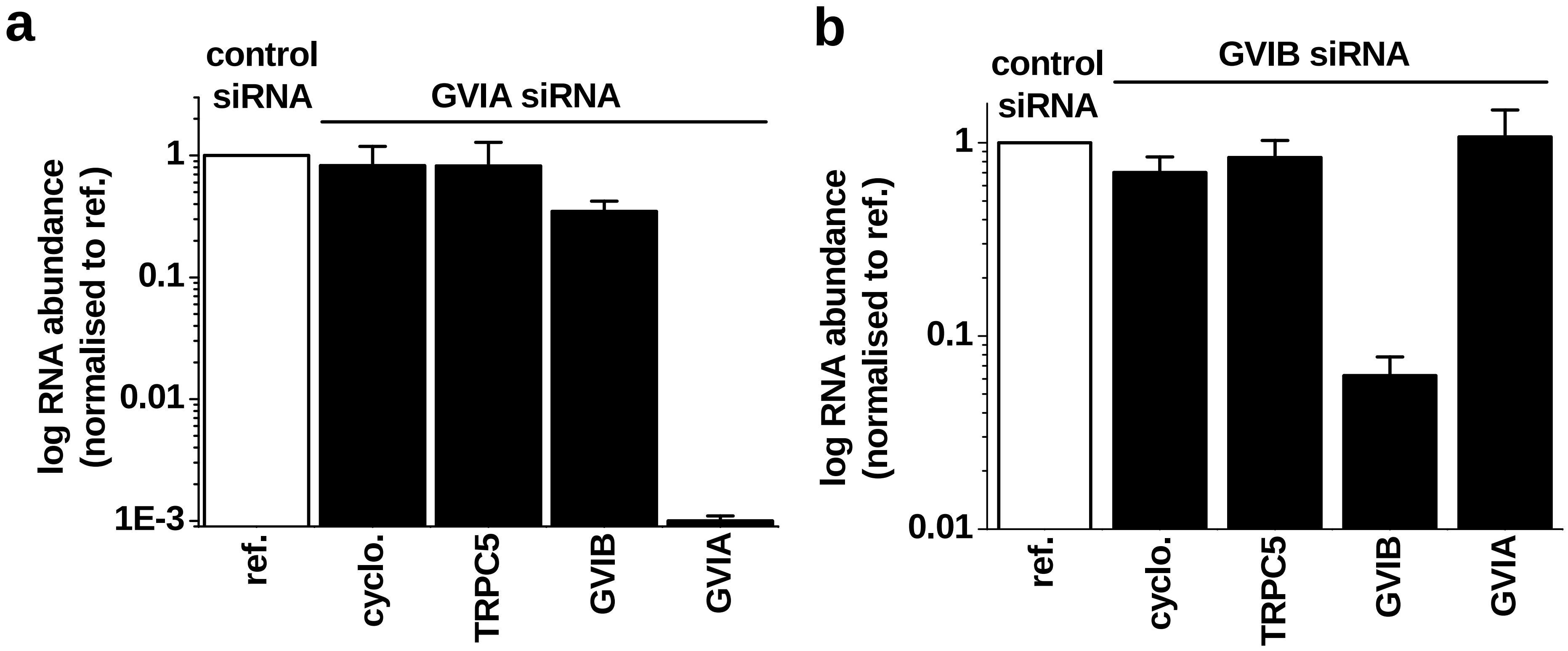

Supplementary Figure II

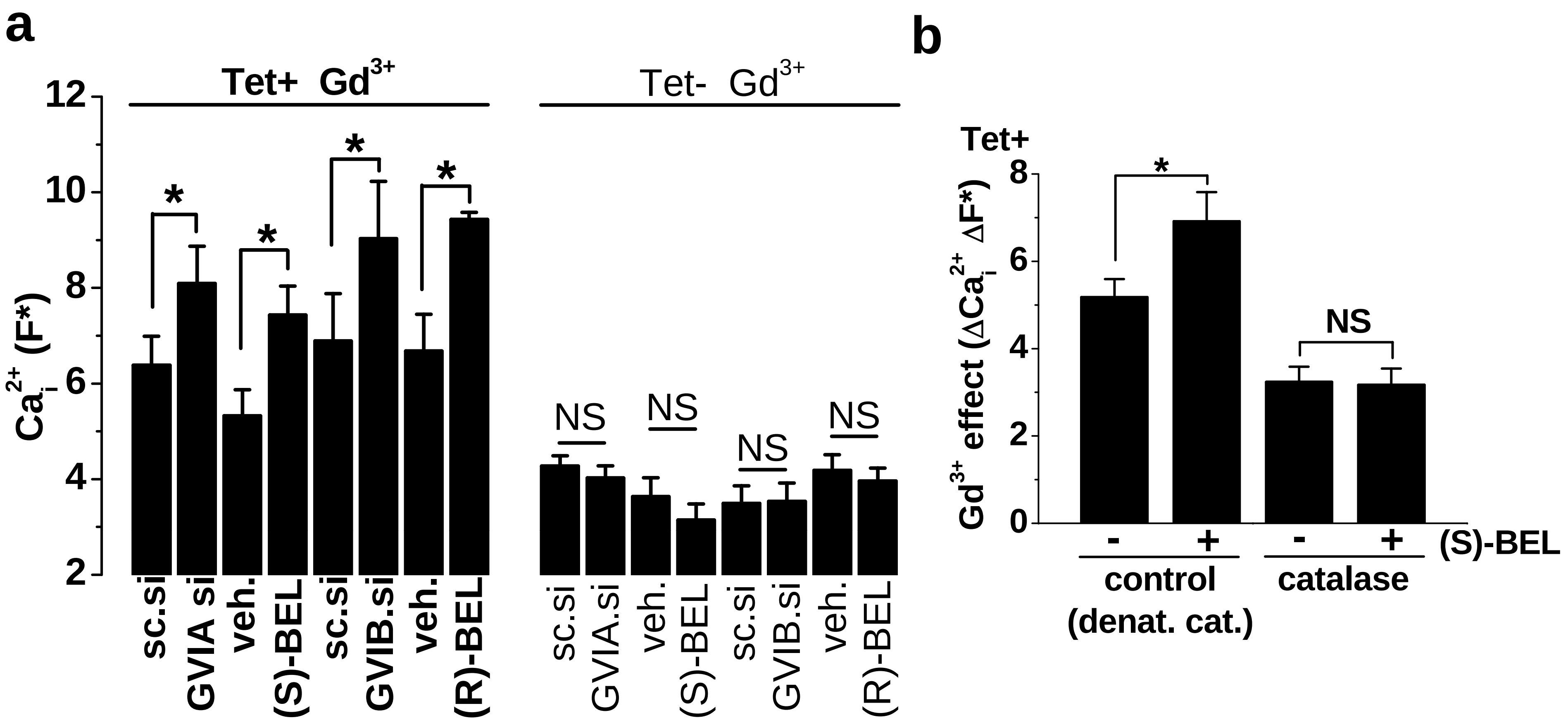

Supplementary Figure III
